# High‐Order Janus Bound States in the Continuum Assisted by Unidirectional Guided Resonances

**DOI:** 10.1002/nap2.70219

**Published:** 2026-07-21

**Authors:** Zengping Su, Jue Li, Haoye Qin, Wenjing Lv, Yongkang Wang, Wei Li, Linhan Lin, Bo Li, Qinghua Song

**Affiliations:** ^1^ Tsinghua Shenzhen International Graduate School Tsinghua University Shenzhen China; ^2^ Department of Electrical and Computer Engineering National University of Singapore Singapore; ^3^ State Key Laboratory of Precision Measurement Technology and Instruments Department of Precision Instrument Tsinghua University Beijing China; ^4^ Suzhou Laboratory Suzhou China

## Abstract

Existing realizations of Janus bound states in the continuum (BICs) rely primarily on manipulating polarization singularities derived from accidental BICs. Here, we introduce a distinct strategy to engineer high‐order Janus BICs by leveraging interband‐coupling‐induced unidirectional guided resonances (UGRs). By breaking the out‐of‐plane symmetry in a square‐lattice photonic crystal slab, we manipulate the momentum‐space evolution of UGRs, driving them to coalesce with an intrinsic symmetry‐protected BIC at the Brillouin zone center. This process gives rise to a high‐order Janus BIC exhibiting extreme radiative asymmetry: it yields an upward‐radiated vortex beam with a topological charge of −3, while maintaining a +1 charge downward. Our findings establish a novel paradigm for tailoring momentum‐space polarization singularities, unlocking new potential for generating vortex beams with direction‐dependent topological charges in advanced optical information processing.

## Graphical abstract

A high‐order Janus bound state in the continuum, featuring a *q* = −3 vortex in the upward radiation channel and a *q* = +1 vortex in the downward channel, is realized in truncated‐cone photonic crystal slabs by merging unidirectional guided resonances with a symmetry‐protected BIC, enabling direction‐dependent control of topological light emission.

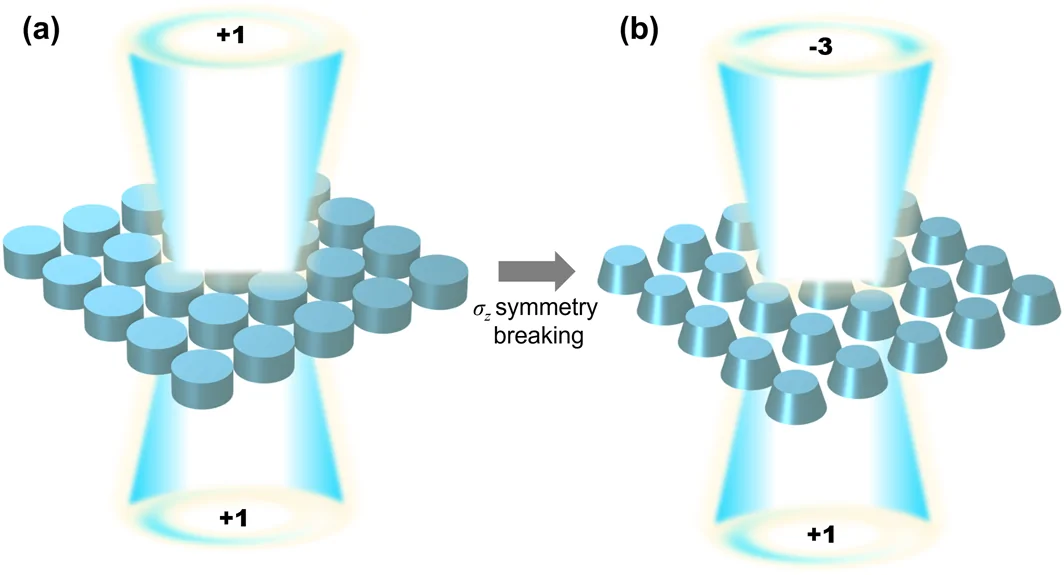

## 1 Introduction

The exploration of radiative polarization textures in the momentum space of photonic crystal slabs (PhCS) has surged recently [1, 2, 3]. These vector fields are fundamentally governed by polarization singularities, which are topologically defined by their conserved topological charges. Bound states in the continuum (BICs) are nonradiative eigenmodes embedded within the radiation continuum, offering theoretically infinite quality (Q) factors [4, 5, 6, 7]. Topologically, these states are identified as polarization singularities carrying integer charges, which manifest as vortex configurations (V‐points) in the momentum‐space polarization vector field [8, 9, 10]. BICs fall into two distinct classes governed by their formation mechanisms: symmetry‐protected and accidental [11]. Symmetry‐protected BICs (SP‐BICs) emerge at high symmetry points in the Brillouin zone, where a symmetry incompatibility completely decouples the eigenmode from the radiation continuum [12, 13, 14, 15, 16]. Conversely, accidental BICs (ABICs) can be engineered at general momentum points through the precise tuning of structural parameters, which induces destructive interference among the radiating waves [17, 18, 19, 20]. The extreme confinement of light fields and the rich polarization textures associated with BICs render them a powerful platform for advanced photonics. These unique properties hold significant promise for diverse applications, including the generation of nonlocal vortex beams [21, 22, 23, 24], the enhancement of optical nonlinearities [25, 26, 27], and the development of low‐threshold lasers [28, 29, 30]. Breaking the in‐plane C2 symmetry triggers a topological transition governed by charge conservation: a BIC splits into a pair of circularly polarized states (C‐points), each carrying a half‐integer topological charge [31, 32]. Conversely, two C‐points of identical charge can coalesce to restore a BIC.

Recently, significant attention has been focused on unidirectional guided resonances (UGRs). These states exhibit BIC‐like behavior exclusively within a single radiation channel, effectively suppressing radiation on one side. Distinct from BICs, UGRs possess finite Q factors and typically necessitate the breaking of out‐of‐plane mirror symmetry [33]. Their generation generally follows two strategies. The first involves breaking in‐plane C2 symmetry, which drives the pair of half‐charge C‐points (originally split from a BIC) to recombine on a specific side [33, 34, 35, 36, 37, 38]. The second strategy, which does not require C2 symmetry breaking, relies on the interband coupling of two orthogonal modes to induce destructive interference in the radiation channel on one side [39, 40]. Related studies on avoided crossings and quasi‐UGR‐to‐UGR transitions have shown that the vertical parity of coupled leaky modes and the redistribution of radiative loss between the two output channels are essential for identifying a genuine UGR rather than merely a generic hybridized resonance [39, 40, 41]. UGRs can be understood as states located at off‐Γ points where radiation on one side behaves as a nonradiative BIC carrying an integer topological charge, whereas radiation on the other side appears as a normal radiative state. To emphasize the asymmetric nature of the topological charges carried by the upward and downward polarization vortices, Janus BICs have been proposed. Their upward and downward radiation channels exhibit BICs carrying different topological charges. Previous research on Janus BICs has introduced out‐of‐plane symmetry breaking through structure or material optical parameters. This causes two pairs of C‐points, split from off‐*Γ* ABICs, to merge with the BIC at the *Γ* point in the upward or downward radiation channel [42, 43, 44]. Consequently, this constructs a Janus BIC where the upward radiation channel carries a BIC with a topological charge of +1, and the downward channel carries a BIC with a topological charge of −1. However, this conventional C‐point merging route inherently limits the resultant singularities to fundamental, low‐order topological charges. The construction of high‐order Janus BICs remains to be systematically explored.

In this work, we report the realization of a high‐order Janus BIC that emits vortex beams with topological charges of *q* = −3 and *q* = +1 into the upward and downward radiation channels, respectively. This is achieved by merging UGRs with a SP‐BIC at the *Γ* point. By breaking the out‐of‐plane symmetry via the transformation of the cylindrical array into a truncated cone structure, we enable interband coupling between the originally orthogonal TE and TM modes along the *Γ*‐X or *Γ*‐M directions. This coupling gives rise to UGRs carrying a topological charge of −1. Because the TM mode hosts a symmetry‐protected BIC with a charge of +1 at the *Γ* point, tuning the structural parameters drives these UGRs toward the *Γ* point where they merge with the central BIC. This merger yields a net topological charge of −3 in the upward radiation channel. We have numerically validated the Janus nature of the proposed structure, demonstrating upward and downward radiated vortex beams with topological charges of −3 and +1, respectively. Overall, these results establish a general strategy for constructing high‐order topological states via UGR merging, offering a flexible platform for advanced topological wave engineering in momentum space.

## 2 Results and Discussion

Figure 1 demonstrates the realization of a high‐order Janus BIC by breaking the out‐of‐plane symmetry of a square‐lattice PhCS. By transforming cylindrical pillars into truncated cones, we create a state with extreme topological asymmetry, characterized by the simultaneous emission of a *q* = −3 vortex beam into the upward channel and a *q* = +1 vortex beam into the downward channel. To realize Janus BICs, we designed a PhCS by embedding a periodic array of truncated cones (*n* = 2.4) within a homogeneous medium (*n* = 1.46). Figure 2a presents the unit cell schematic of truncated cones, displaying the top view (*x*–*y* plane) on the left and the cross‐section (*x*–*z* plane) on the right. The lattice constant is set to *a* = 400 nm, whereas the cone geometry is defined by its base diameter *D* (= 0.8*a*), height *H*, and sidewall inclination angle *θ*. Figure 2b depicts the first Brillouin zone and the in‐plane field profiles of the TM_A_, TE_B_, and TE_C_ eigenmodes at the *Γ* point. The complex eigenfrequencies of eigenmodes are calculated using COMSOL Multiphysics. Periodic boundary conditions are applied in the *x* and *y* directions, while perfectly matched layers are set in the *z* direction to absorb radiation. The TM_A_ mode supported by the square lattice of cylindrical pillars manifests as an SP‐BIC with a topological charge of +1 at the *Γ* point. This is evidenced by its theoretically infinite Q factor at *Γ* point and the polarization vortex texture in the vicinity of the *Γ* point (see Supporting Information S1: Figure S1). TE_B_ and TE_C_ modes constitute a degenerate pair that remains strictly orthogonal to the TM_A_ mode. Figure 2c reveals the *x*–*z* field profiles of the TM_A_ and TE_B_ modes along the *Γ*‐X path. The *x*–*z* field distributions indicate that the degenerate TE_B_ and TE_C_ branches originate from folded fundamental TE‐like guided modes of the corresponding homogeneous slab. In the extended‐zone band‐folding convention, this degeneracy corresponds to the first folded meeting at Γ, namely the second‐order *Γ* point, rather than to higher‐order *Γ*‐point degeneracies [45]. At *θ* = 0°, the preservation of vertical mirror symmetry dictates that TM_A_ and TE_B_ exhibit odd and even parities along the *z*‐axis, respectively; this strict orthogonality prevents any interband coupling. Conversely, setting *θ* = 15.27° breaks this symmetry, enabling interband coupling between the two modes. This interaction induces destructive interference in the upward radiation channel, ultimately leading to the formation of a UGR. Analogously, along the *Γ*‐M direction, out‐of‐plane symmetry breaking triggers interband coupling between the TM_A_ and TE_C_ modes, likewise enabling the formation of a UGR.

**FIGURE 1:**
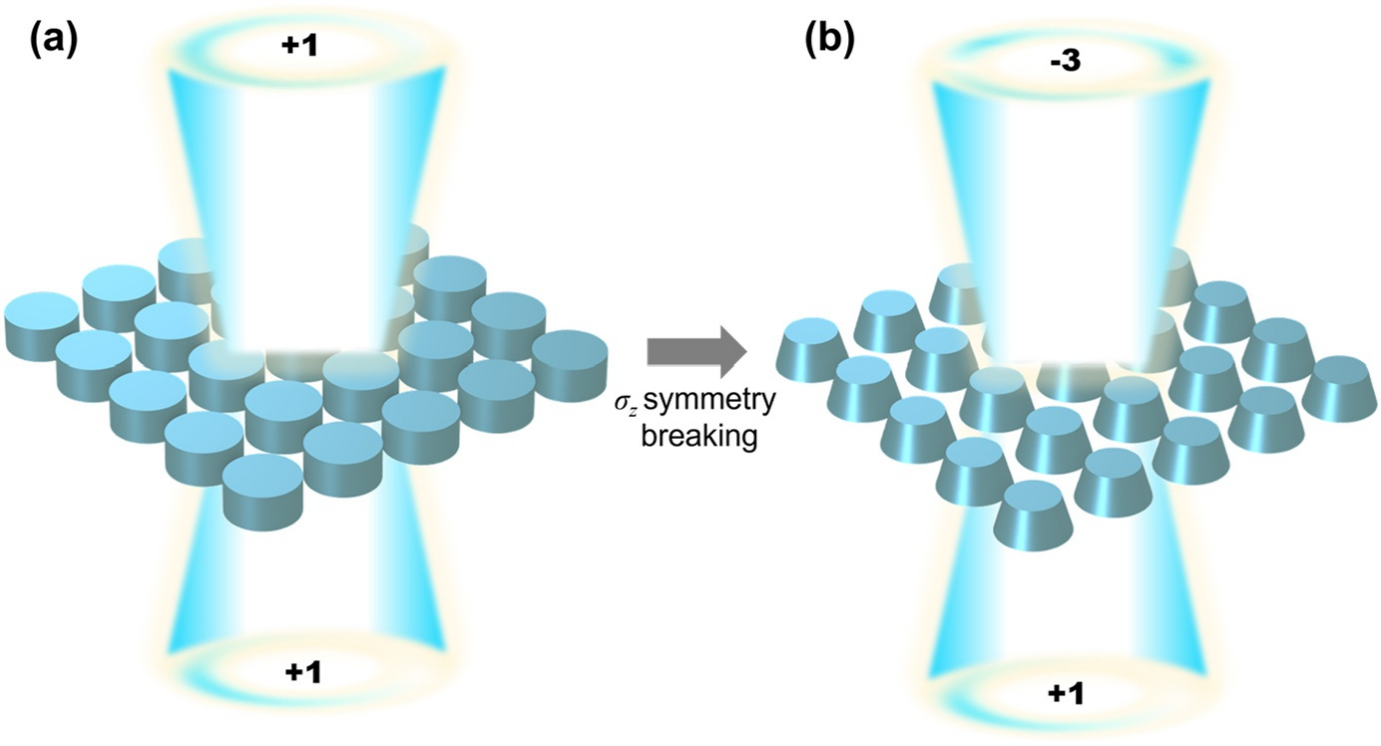
Schematic illustration of realizing high‐order Janus BICs by breaking the *σ*_*z*_ symmetry. (a) A symmetric square‐lattice array of cylindrical pillars radiates upward and downward vortex beams induced by a SP‐BIC, both carrying identical topological charges of *q* = +1. (b) Introducing a sidewall inclination transforms the cylinders into truncated cones, realizing a high‐order Janus BIC where the radiated upward and downward vortex beams carry distinct topological charges of *q* = −3 and *q* = +1, respectively.

**FIGURE 2:**
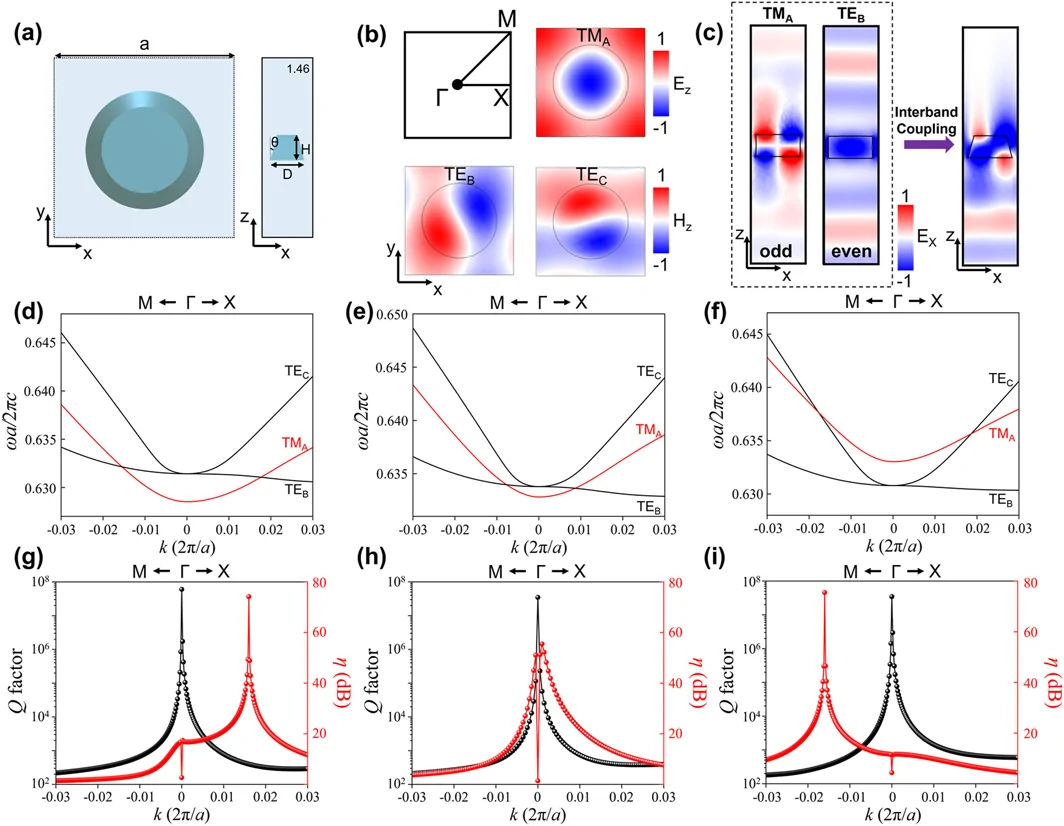
(a) Schematic of a square‐lattice array of truncated cones. Top view (*x*–*y* plane) and cross‐sectional view (*x*–*z* plane) of the truncated cone unit cell. (b) Schematic of the first Brillouin zone and the in‐plane (*x*–*y*) field profiles of the TM_A_, TE_B_, and TE_C_ modes at the *Γ* point. (c) Cross‐sectional (*x*–*z*) field profiles illustrating the formation of a UGR induced by interband coupling between TM_A_ and TE_B_ modes at an off‐*Γ* momentum along the *Γ*‐X direction, after out‐of‐plane symmetry breaking. Normalized frequency dispersions of TM_A_, TE_B_, and TE_C_ modes along the M‐Γ‐X path for three structural configurations: (d) *H* = 0.40033a, *θ* = 15.27°; (e) *H* = 0.3785a, *θ* = 16.03°; and (f) *H* = 0.3729a, *θ* = 13.375°. (g–i) Corresponding Q factors and radiation asymmetry ratios (*η*) of the TM_A_ mode along the M‐Γ‐X path for the same parameter sets as in d–f.

To model this interaction, we consider the two orthogonal eigenmodes, TM_A_ and TE_B_, with their respective complex eigenfrequencies *Ω*_1_ (= *ω*_*1*_ − *iγ*_*1*_) and *Ω*_2_ (= *ω*_*2*_ − *iγ*_*2*_). With the out‐of‐plane symmetry broken by a tilted sidewall angle *θ*, the system in the vicinity of the TM_A_‐TE_B_ band crossing point is described by the Hamiltonian [39]:

(1) $$H=\left[\begin{array}{cc} \omega_{0}+i\gamma_{1}+\nu_{g1}\Delta k_{\|} & \kappa_{12}\left(\Delta k_{\|}\right)\tan\;\theta \\ \kappa_{21}\left(\Delta k_{\|}\right)\tan\;\theta & \omega_{0}+i\gamma_{2}+\nu_{g2}\Delta k_{\|} \end{array}\right].$$

Here, *ω*_0_ is the central frequency at the crossing point; Δ*k*_∥_ is the in‐plane momentum displacement; *γ*_1,2_ and *v*_g1,2_ are the decay rates and group velocities of the uncoupled modes *Ω*_1_ and *Ω*_2_; *κ*_12_ and *κ*_21_ are the interband coupling coefficients. The eigenvalues corresponding to the Hamiltonian in Equation (1) are given by

(2) $$\lambda_{\pm }=\omega_{0}+i\frac{\gamma_{1}+\gamma_{2}}{2}+\frac{v_{g1}-v_{g2}}{2}\pm \sqrt{\frac{{\left[i\left(\gamma_{1}-\gamma_{2}\right)+\left(v_{g1}+v_{g2}\right)\Delta k_{\|}\right]}^{2}}{4}+\kappa_{12}\kappa_{21}{\left(\tan\;\theta \right)}^{2}}.$$

Thus, the condition for complete degeneracy of the eigenvalues for the two coupled modes is given by

(3) $$4\kappa_{12}\left(k_{\|}\right)\kappa_{21}\left(k_{\|}\right){\left(\tan\;\theta_{\text{EP}}\right)}^{2}=-{\left[i\left(\gamma_{1}-\gamma_{2}\right)+\left(v_{g1}+v_{g2}\right)\Delta k_{\|}\right]}^{2}.$$

The interband coupling strength, which increases with the sidewall tilt angle *θ* because of reduced mode orthogonality, can be categorized into two distinct regimes separated by a critical angle *θ*_EP_: a weak coupling regime (*θ* < *θ*_EP_) and a strong coupling regime (*θ* > *θ*_EP_). This distinction dictates the eigenfrequency behavior near the band crossing, as predicted by Equation (2). In this work, the interband coupling mechanisms employed for UGR generation operate within the weak coupling regime. This regime is characterized by the crossing of the real parts of the eigenfrequencies and the anti‐crossing of their imaginary parts, as shown in Supporting Information S1: Figure S4.

Although Equation (1) captures the interband coupling and the associated avoided‐crossing behavior, the directional radiation characteristic of the UGR can be further understood by examining the radiative coupling of the hybridized eigenmodes [41]. For the two interacting eigenmodes with complex frequencies *Ω*_1_ and *Ω*_2_, the radiation coupling matrix can be written as follows:

(4) $$\begin{array}{c} D=\left(\begin{array}{cc} d_{1u} & d_{2u}\\ d_{1d} & d_{2d} \end{array}\right). \end{array}$$

where *d*_*ju*_ and *d*_*jd*_ denote the radiation coupling coefficients of mode *j* into the upward and downward radiation channels, respectively. According to energy conservation and time‐reversal symmetry, the coupling matrix satisfies $D^{†}D=2\Gamma ,$ which leads to

(5) $$\begin{array}{c} |d_{1u}{|}^{2}\;+\;|d_{1d}{|}^{2}=2\gamma_{1u}+2\gamma_{1d}, \end{array}$$

(6) $$\begin{array}{c} |d_{2u}{|}^{2}\;+\;|d_{2d}{|}^{2}=2\gamma_{2u}+2\gamma_{2d}, \end{array}$$

(7) $$\begin{array}{c} \left|d_{1u}\right|\left|d_{2u}\right|e^{i\left(\theta_{1u}-\theta_{2u}\right)}+\left|d_{1d}\right|\left|d_{2d}\right|e^{i\left(\theta_{1d}-\theta_{2d}\right)}=2\beta . \end{array}$$

where *θ*_*ju*_ and *θ*_*jd*_ are the radiation phases of mode *j* in the upward and downward channels, respectively, whereas *γ*_*ju*_ and *γ*_*jd*_ represent the corresponding radiative decay rates. Following the treatment in temporal coupled‐mode theory, the radiative coupling coefficients can be written as, $\left|d_{ju}\right|=\sqrt{2\gamma_{ju}}$ and $\left|d_{jd}\right|=\sqrt{2\gamma_{jd}}$. Substituting these relations into Equation (7), the far‐field coupling coefficient becomes

(8) $$\begin{array}{c} \beta =\sqrt{\gamma_{1u}\gamma_{2u}}e^{i\left(\theta_{1u}-\theta_{2u}\right)}+\sqrt{\gamma_{1d}\gamma_{2d}}e^{i\left(\theta_{1d}-\theta_{2d}\right)}. \end{array}$$

For a vertically symmetric structure, the phase relations in the upward and downward channels are identical. In contrast, the tilted sidewall breaks the out‐of‐plane mirror symmetry and causes the phase differences in the two channels to become unequal, $\theta_{1u}-\theta_{2u}\neq \theta_{1d}-\theta_{2d}$. As a consequence, the radiative fields associated with the two hybridized modes interfere differently in the upward and downward radiation channels. Under appropriate symmetry‐breaking conditions, destructive interference suppresses radiation into one direction, whereas radiation in the opposite direction remains strong. This directional radiation imbalance gives rise to the unidirectional guided resonance observed in our structure.

Figure 2d–i map the evolution of UGRs along the M‐Γ‐X high‐symmetry path, driven by interband coupling under three distinct structural configurations. To quantitatively evaluate the directionality of the radiation, we define an asymmetry ratio (*η*) as the ratio of the downward radiative loss to the upward radiative loss in momentum space. In our numerical model, this is equivalently calculated by *η* = P_down_/P_up_, where P_up_ and P_down_ represent the total far‐field radiated powers extracted from two monitor planes located at a certain distance above and below the PhCS, respectively. With this definition, a large *η* indicates suppressed upward radiation, whereas *η* approaching zero indicates suppressed downward radiation. Figure 2d–f plots the normalized band structures, while Figure 2g–i depicts the corresponding Q factors and *η* specifically for the TM_A_ mode. The three cases correspond to specific parameter sets: *H* = 0.40033*a* with *θ* = 15.27°; *H* = 0.3785*a* with *θ* = 16.03°; and *H* = 0.3729*a* with *θ* = 13.375°. The existence of both the BIC and UGRs is corroborated by the calculated Q factor and *η* of the TM_A_ band along the M‐Γ‐X high‐symmetry lines. The Q factor distributions across all three parameter sets confirm the persistence of an SP‐BIC at the *Γ* point, which remains robust against out‐of‐plane symmetry breaking. Figure 2d,g illustrates interband coupling between TM_A_ and TE_B_ along *Γ*‐X, creating a UGR (*η* = 74.16) at *k* = 0.016. Further tuning of *H* and *θ* drives this UGR toward the *Γ* point, leading to their merger (Figure 2e,h). Subsequently, parameter adjustments enable coupling between TM_A_ and TE_C_ along *Γ*‐M direction, generating a UGR (*η* = 75.46) at *k* = −0.016. These results demonstrate that interband coupling can be selectively induced with either TE_B_ or TE_C_ mode, and the resulting UGR position is tunable along high‐symmetry lines. Consequently, merging a UGR with the SP‐BIC at *Γ* point is a viable strategy.

To better understand the formation and evolution of UGRs, we next analyze the far‐field polarization distributions in momentum space for both the upward and downward radiation channels of the TM_A_ mode. Here, the far‐field polarization vector is defined as follows [10]:

(9) $$c\left(k_{∥}\right)=\left[c_{x}\left(k_{∥}\right),c_{y}\left(k_{∥}\right),c_{z}\left(k_{∥}\right)\right]=\frac{\iint_{\text{unit}}e^{ik_{x}x+ik_{y}y}E\left(x,y,z\right)dxdy}{\iint_{\text{unit}}dxdy},$$

where the domain of integration is a single unit cell within an *x*–*y* plane positioned either above or below the PhCS. Although the polarization vector is inherently three‐dimensional, its topological features can be analyzed by considering its projection onto the *x*–*y* plane. The Stokes parameters (*S*_0_, *S*_1_, *S*_2_, *S*_3_) describing the 2D polarization state of the far field for a given in‐plane wavevector *k*_∥_ are defined as $S_{0}={\left|c_{x}\left(k_{∥}\right)\right|}^{2}+{\left|c_{y}\left(k_{∥}\right)\right|}^{2}$, $S_{1}={\left|c_{x}\left(k_{∥}\right)\right|}^{2}-{\left|c_{y}\left(k_{∥}\right)\right|}^{2}$, $S_{2}=2\text{Re}\left[c_{x}^{*}\left(k_{∥}\right)c_{y}\left(k_{∥}\right)\right]$, and $S_{3}=2\text{Im}\left[c_{x}^{*}\left(k_{∥}\right)c_{y}\left(k_{∥}\right)\right]$. The orientation of the polarization ellipse is described by the angle *ϕ*(*k*_∥_), defined as $\phi \left(k_{∥}\right)=\frac{1}{2}\arg\left[S_{1}+iS_{2}\right]$, where it represents the angle of the polarization ellipse's major axis with respect to the *x*‐axis.

To explicitly verify the asymmetric topological nature of the high‐order Janus BIC, we investigate the far‐field polarization textures for the upward and downward radiation channels separately. Figure 3 illustrates the evolution of the upward‐radiating polarization field and *η* in momentum space, whereas the downward‐radiating counterparts are provided in Supporting Information S1: Figure S2. To quantify the topological properties of the BICs and UGRs, we define the topological charge (*q*) as the winding number of the far‐field polarization vector in momentum space: $q=\frac{1}{2\pi }\oint_{C}{dk}_{∥}\cdot \nabla_{k_{∥}}\phi \left(k_{∥}\right)$, where *C* denotes a closed loop encircling the polarization singularity in a counterclockwise direction. Figure 3a–f map the polarization states and the orientation angle *ϕ*(*k*_∥_) of the polarization ellipses, respectively. Initially, for the parameters *H* = 0.40033*a* and *θ* = 15.27° (Figure 3a,d), the upward radiation field features four UGRs, each carrying a topological charge of *q* = −1, distributed along the *Γ*‐X and *Γ*‐Y directions. These UGRs coexist with a central SP‐BIC (*q* = +1) at the *Γ* point. At *H* = 0.3785*a* and *θ* = 16.03°, the four off‐Γ UGRs converge to the *Γ* point, merging with the pre‐existing BIC to synthesize a high‐order BIC with a topological charge of *q* = −3. This topological signature is explicitly captured in Figure 3e: Tracing a counterclockwise loop around the singularity reveals that the orientation angle *ϕ*(*k*_∥_) of the polarization ellipses undergoes six transitions from π/2 to −π/2. Applying the topological charge formula, the total winding accumulation corresponds precisely to a net charge of *q* = −3. Subsequently, further adjustment to *H* = 0.3729*a* and *θ* = 13.375° causes the four UGRs to split and migrate away from the *Γ* point along the *Γ*‐M direction to coordinates (± 0.016, ± 0.016). Throughout this process, the SP‐BIC (*q* = +1) persists at the *Γ* point. The corresponding normalized frequency and Q factor distributions are detailed in Supporting Information S1: Figure S3. Finally, Figure 3g–i demonstrate that this UGR evolution can be visualized intuitively through the momentum‐space distribution of *η*.

**FIGURE 3:**
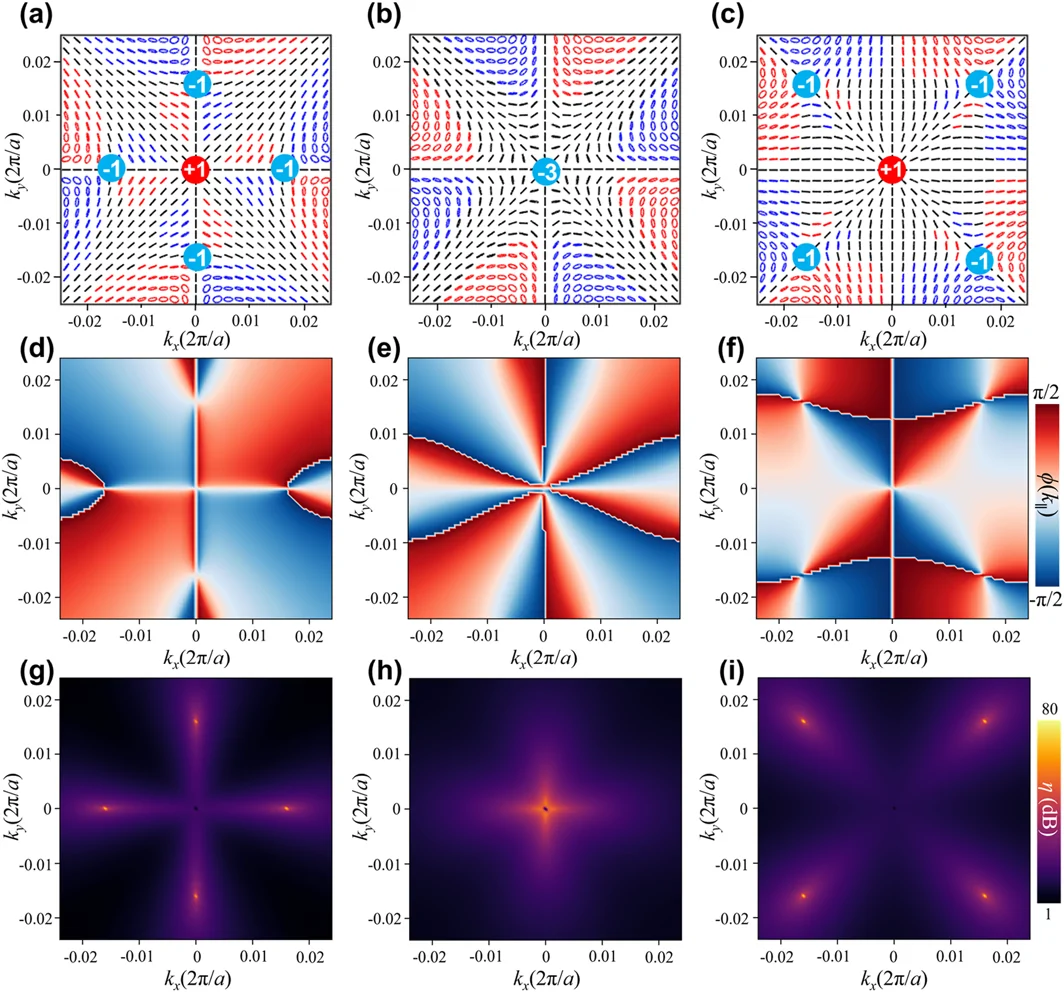
Momentum‐space far‐field polarization textures for upward radiation under structural parameters: (a) *H* = 0.40033*a*, *θ* = 15.27°; (b) *H* = 0.3785*a*, *θ* = 16.03°; and (c) *H* = 0.3729*a*, *θ* = 13.375°. (d–f) Corresponding distributions of the polarization ellipse orientation angle *ϕ*(*k*_∥_) for the configurations in a–c. (g–i) Momentum‐space maps of the radiation asymmetry ratio (*η*) for the same parameter sets as in a–c.

Finally, to confirm the high‐order Janus BIC realization and demonstrate its utility, we present the radiated vortex emissions from the upward and downward channels. First, the circularly polarized scattering parameters near the *Γ* point are calculated using COMSOL Multiphysics for a single unit cell under periodic boundary conditions, establishing the dependence of the active modal response on the in‐plane wavevector *k*_∥_. To excite the resonant mode associated with the Janus BIC, a set of electric dipoles is placed inside the photonic crystal slab. Subsequently, the near‐field distribution of a finite‐size resonant cavity under lasing conditions is reconstructed by interpolating the momentum‐space scattering parameters weighted by the transverse wavevector spectrum of a Gaussian field. The far‐field characteristics of the vortex laser emission are obtained through a standard Huygens near‐to‐far‐field transformation, which propagates the reconstructed near‐field into the radiation zone. The upward and downward radiations are then collected on two parallel observation planes, yielding the corresponding real‐space intensity and phase distributions. Figure 4a,b displays the real‐space intensity distributions for the upward and downward emissions, respectively, both exhibiting the characteristic doughnut‐shaped profiles. Correspondingly, Figure 4c,d reveals their spiral phase distribution in the real space. The combination of Figure 4a,c clearly identifies the upward emission as a vortex beam carrying a topological charge of *q* = −3, whereas Figure 4b,d confirms the downward emission as a vortex beam with *q* = +1. These distinct topological signatures successfully validate the existence of the high‐order Janus BIC.

**FIGURE 4:**
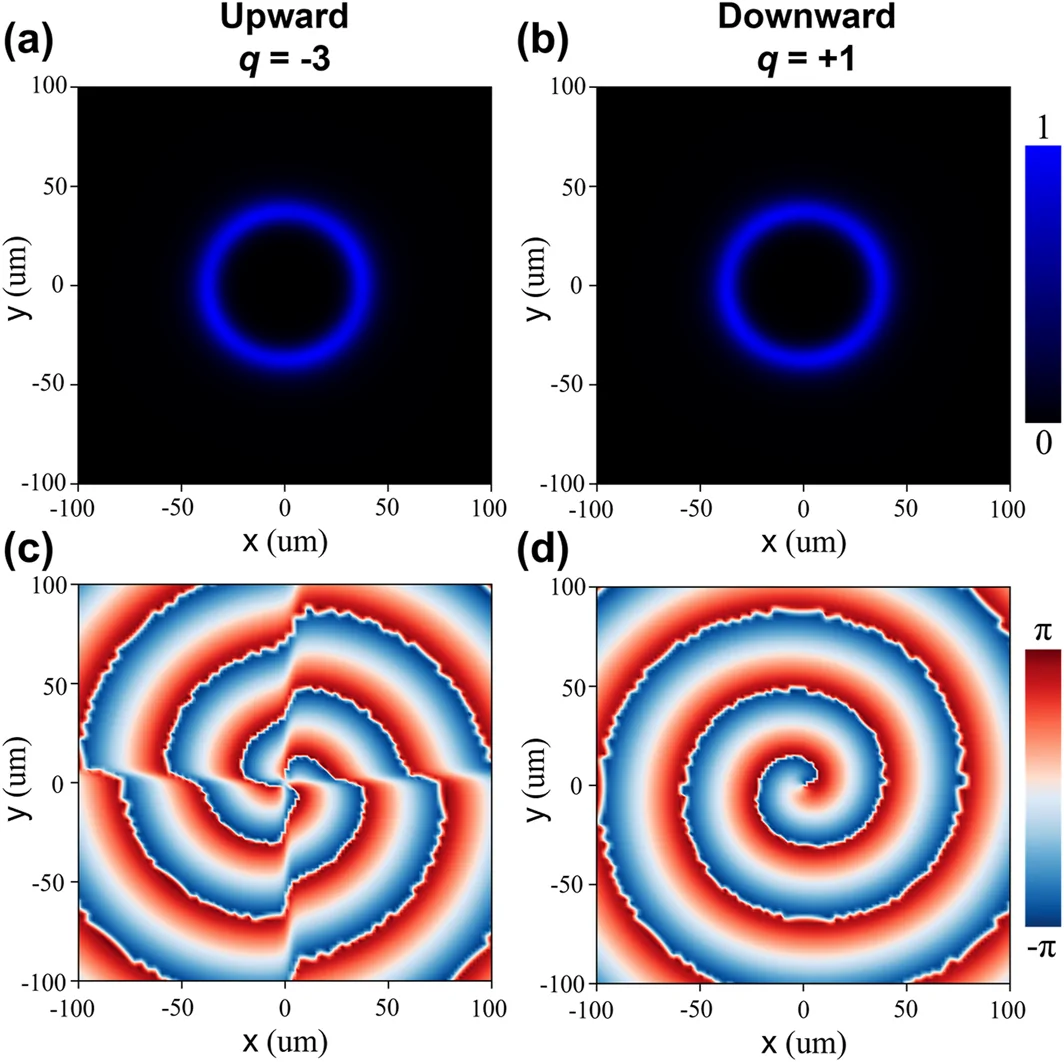
Real‐space intensity distributions of the upward (a) and downward (b) radiated vortex beams for the structural parameters *H* = 0.3785*a*, *θ* = 16.03°. Corresponding spiral phase distributions for the upward (c) and downward (d) radiation channels.

The two radiation channels are intrinsically connected through the geometric nature of the truncated‐cone unit cell, where a vertical inversion directly interchanges the upward and downward channels while preserving the modal characteristics, as shown in Supporting Information S1: Figure S5. As a result, their associated topological charges are correspondingly reversed, establishing a one‐to‐one mapping between the two channels. Beyond structural inversion, a complementary degree of freedom arises from modal selection: distinct eigenmodes within the truncated‐cone geometry (with a larger bottom and smaller top) can support UGRs with different topological charges. In particular, another pair of modes supports UGRs with a charge of +1, which, following the same design principle, enables a vortex configuration with a +3 charge in the downward channel and a −1 charge in the upward channel, as shown in Supporting Information S1: Figure S6. These results demonstrate that the radiation topology in the two channels can be flexibly and independently engineered.

## 3 Conclusion

In conclusion, we have theoretically proposed and numerically demonstrated a new strategy to engineer high‐order Janus BICs in PhCS. By breaking the out‐of‐plane symmetry via the transformation of cylindrical pillars into truncated cones in a square‐lattice array, we enabled the formation of UGRs via interband coupling between orthogonal eigenmodes. We demonstrated that through precise manipulation of geometric parameters, UGRs initially located away from the Brillouin zone center can be driven to coalesce with the intrinsic symmetry‐protected BIC at the *Γ* point. This topological fusion results in the formation of a unique high‐order Janus BIC exhibiting extreme radiative asymmetry: it emits a vortex beam with a topological charge of −3 into the upward channel, whereas the downward radiation retains a topological charge of +1. Furthermore, we utilized the momentum‐space distribution of the radiation asymmetry ratio *η* to quantitatively track the evolution and merging of these topological singularities. These results establish a deterministic strategy for synthesizing complex topological charges via UGR coalescence, significantly expanding the toolkit for manipulating polarization singularities in momentum space. The direction‐dependent topological charges may be useful for compact generation of channel‐dependent orbital‐angular‐momentum beams, directional surface emitters, and chiral or topological photonic interfaces in which the upward and downward radiation channels perform different optical functions.

## Author Contributions

Z.S. and Q.S. conceived the project. Z.S. and J.L. carried out numerical simulation. Z.S. and Q.S. wrote the manuscript. Z.S., H.Q. and Q.S. revised the manuscript. Q.S. supervised the entire project. All authors discussed the results and commented on the article.

## Funding

Q.S. acknowledges the funding support from the National Key R&D Program of China (Grant 2023YFB3811400), the National Natural Science Foundation of China (Grant 12474388), Guangdong Basic and Applied Basic Research Foundation (Grant 2025A1515011483), Shenzhen Science and Technology Program (Grant JCYJ20230807111706014). This work is also funded by the Basic Research Program of Jiangsu (Grant BK20243029). H.Q. acknowledges the funding support from Swiss National Science Foundation (Grant 239281).

## Conflicts of Interest

The authors declare no conflicts of interest.

## Supporting information


Supporting Information S1

## Data Availability

The data that support the findings of this study are available from the corresponding author upon reasonable request.
