# Supplementary material for: High‐Order Janus Bound States in the Continuum Assisted by Unidirectional Guided Resonances
Source: Nanophotonics. 2026 Jul 21;15(14):e70219. doi: 10.1002/nap2.70219 (PMC13642067; doi:10.1002/nap2.70219)
Supplement: Supplementary file 1 [file NAP2-15-e70219-s001.docx]

Supporting Information for

High-order Janus bound states in the continuum assisted by unidirectional guided resonances

Zengping Su^1#^, Jue Li^1#^, Haoye Qin^2#^, Wenjing Lv^1^, Yongkang Wang^1^, Wei Li^1^,

Linhan Lin^3^, Bo Li^1,4^, Qinghua Song^1,4*^

*^1^Tsinghua Shenzhen International Graduate School, Tsinghua University, Shenzhen 518055, China*

*^2^Department of Electrical and Computer Engineering, National University of Singapore, Singapore 117583, Singapore*

*^3^State Key Laboratory of Precision Measurement Technology and Instruments, Department of Precision Instrument, Tsinghua University, Beijing 100190, China*

*^4^Suzhou Laboratory, Suzhou 215000, China*

*^#^These authors contributed equally to this work: Zengping Su*, *Jue Li*, *Haoye Qin*

*^*^Corresponding Authors: [song.qinghua@sz.tsinghua.edu.cn](mailto:song.qinghua@sz.tsinghua.edu.cn)*

Figure S1. Characteristics of the symmetry-protected BIC in a cylindrical array. (a) Schematic of the PhCS with cylindrical pillars. (b) Cross-sectional view (*x*-*z* plane) of the unit cell with cylinder height *H* = 0.4*a* and diameter *D* = 0.8*a*, alongside the field profile of the TM_A_ mode at the Γ point. (c) Momentum-space distributions of the normalized frequency and quality factor for the TM_A_ mode. (d) Polarization vortex distribution of the TM_A_ mode in momentum space, carrying a topological charge of +1.

Figure S2. Momentum-space far-field polarization textures for downward radiation under structural parameters: *H* = 0.40033*a*, *θ* = 15.27° (a); *H* = 0.3785*a*, *θ* = 16.03° (b); and *H* = 0.3729*a*, *θ* = 13.375° (c). Corresponding distributions of the polarization ellipse orientation angle *ϕ*(*k*_∥_) for the configurations in a (d), b (e), and c (f).

Figure S3. Momentum-space distributions of the normalized frequency for the structural parameters: *H* = 0.40033*a*, *θ* = 15.27° (a); *H* = 0.3785*a*, *θ* = 16.03° (b); and *H* = 0.3729*a*, *θ* = 13.375° (c). Corresponding Q factors distributions for the same parameter sets as in a (d), b (e), and c (f).

Figure S4. Complex eigenfrequency spectra of the coupled modes demonstrating the weak coupling regime. **(a, d)** The real parts (a) and imaginary parts (d) of the eigenfrequencies for the TM_A_ and TE_B_ modes along the $\Gamma$-X high-symmetry direction. **(b, e)** The real parts (b) and imaginary parts (e) of the eigenfrequencies for the TM_A_ and TE_B_ modes along the $\Gamma$-X high-symmetry direction. **(c, f)** The real parts (c) and imaginary parts (f) of the eigenfrequencies for the TM_A_ and TE_C_ modes along the M-$\Gamma$ high-symmetry direction. All cases clearly exhibit the crossing of the real parts and the distinct anti-crossing of the imaginary parts, which unambiguously characterizes the interband coupling operating in the weak coupling regime.

Figure S5. Demonstration of the construction of UGRs in the opposite radiation channel. (a) Structural inversion of the truncated-cone unit cell and the corresponding reversal of the radiation-channel topology. (b) Cross-sectional (*x*–*z*) field profiles showing the formation of a UGR induced by interband coupling between the TM_A_ and TE_B_ modes at an off-Γ momentum along the Γ–X direction. Real-space intensity and spiral phase distributions of the radiated vortex beams for the upward (c) and downward (d) channels.

Figure S6. Formation of a downward UGR arising from a different eigenmode (*H* = 0.33941*a*, *θ* = 80.66°) and its associated momentum-space radiation properties. Momentum-space far-field polarization textures and distributions of the polarization ellipse orientation angle *ϕ*(*k_∥_*) for the upward (a) and downward (b) radiation channels. (c) Momentum-space map of the radiation asymmetry ratio *η*, highlighting the unidirectional emission into the downward channel. (d) Cross-sectional (*y*–*z*) field profiles showing the formation of the UGR induced by interband coupling between the TM and TE modes at an off-Γ momentum along the Γ–X direction.
